# Reduced Graphene Oxide-Based Double Network Polymeric Hydrogels for Pressure and Temperature Sensing

**DOI:** 10.3390/s18093162

**Published:** 2018-09-19

**Authors:** Wei Liu, Xiaoyuan Zhang, Gang Wei, Zhiqiang Su

**Affiliations:** 1 State Key Laboratory of Chemical Resource Engineering, Beijing University of Chemical Technology, Beijing 100029, China; viviwliu@163.com; 2 Beijing Key Laboratory of Advanced Functional Polymer Composites, Beijing University of Chemical Technology, Beijing 100029, China; 3 Otto Schott Institute of Materials Research, Friedrich-Schiller University Jena, 07743 Jena, Germany; 4 Faculty of Production Engineering, University of Bremen, D-28359 Bremen, Germany

**Keywords:** hydrogels, reduced graphene oxide, temperature, pressure, sensing

## Abstract

We demonstrate the fabrication of novel reduced graphene oxide (rGO)-based double network (DN) hydrogels through the polymerization of poly(*N*-isopropylacrylamide) (PNIPAm) and carboxymethyl chitosan (CMC). The facile synthesis of DN hydrogels includes the reduction of graphene oxide (GO) by CMC, and the subsequent polymerization of PNIPAm. The presence of rGO in the fabricated PNIPAm/CMC/rGO DN hydrogels enhances the compressibility and flexibility of hydrogels with respect to pure PNIPAm hydrogels, and they exhibit favorable thermoresponsivity, compressibility, and conductivity. The created hydrogels can be continuously cyclically compressed and have excellent bending properties. Furthermore, it was found that the hydrogels are pressure- and temperature-sensitive, and can be applied to the design of both pressure and temperature sensors to detect mechanical deformation and to measure temperature. Our preliminary results suggest that these rGO-based DN hydrogels exhibit a high potential for the fabrication of soft robotics and artificially intelligent skin-like devices.

## 1. Introduction

Hydrogels—wet and soft materials that consist of water and cross-linked three-dimensional (3D) networks—have been proven to possess several unique properties, such as stimuli response, shock absorption, swelling, and conductivity [1,2,3]. The created smart hydrogels can be utilized in various fields, including photochemistry, sensors, tissue engineering, biomedical engineering, and materials science [4,5,6]. However, some challenges regarding the synthesis and application of various hydrogels need to be overcome. For instance, hydrogels are mostly brittle, with a fracture energy of 1–10 J/m^2^ and an elastic modulus of around 10 kPa [7,8]. Both values are much lower than those of cartilage in the body—which has a fracture energy of 16 J/m^2^ [9] and an elastic modulus of around 0.32 GPa [10]. 

The formation of double network (DN) hydrogels is one of the potential ways to significantly improve the mechanical properties of hydrogels. For instance, Gong and co-workers developed DN hydrogels (PAMPS-PAAm) including poly(2-acrylamido-2-methylpropanesulfonic acid) (PAMPS) and polyacrylamide (PAAm) with enhanced mechanical strength [11,12]. The fabricated DN hydrogels exhibited fracture toughness, fracture tensile stress, and fracture tensile strain of 10^2^–10^3^ J/m^2^, 1–10 MPa, and 1000–2000%, respectively. It is well-known that the promising flexibility of hydrogels comes from a few factors, such as the combination of brittle PAAm and ductile PAMPS networks, higher molar content of the ductile polymer, and asymmetric gel structure [13,14,15,16]. 

Based on these principles of the two-step polymerization method, various tough DN hydrogels have been fabricated, such as the microgel-reinforced DN gel [14], void DN gel [17], and inverse DN gel [18]. For example, Huang et al. prepared a novel inorganic–organic DN hydrogel by using graphene and poly(acrylic acid) (PAA) hydrogel. The use of graphene made it possible to fabricate a 3D structure with a tightly cross-linked network [19]. In addition, the acrylic acid monomer dispersed in the graphene structure and cross-linked into the second ductile network. This novel DN hydrogel showed great elasticity and electrical conductivity. However, the complex and rigorous fabrication processes and the acid residue limited the application of the fabricated DN hydrogels in the field of sensors. 

Herein, we developed a facile and efficient method to construct organic–organic DN hydrogels by using reduced graphene oxide (rGO), poly(*N*-isopropylacrylamide) (PNIPAm), and carboxymethyl chitosan (CMC) together. The thermosensitive PNIPAm serves as the first tightly cross-linked network, and the CMC/rGO acts as the second ductile network, in which the GO nanosheets are reduced to rGO by CMC via the amidation reaction. In addition, the dense PNIPAm and loose CMC/rGO networks are physically connected by hydrogen bonds. It was found that the obtained PNIPAM/CMC/rGO DN hydrogels had potential applications in both pressure and temperature sensing. 

## 2. Materials and Methods

### 2.1. Materials

*N*-isopropylacrylamide (NIPAm), *N*,*N*’-methylenebisacrylamide (BIS), ammonium persulfate (APS), and *N*,*N*,*N*’,*N*’-tetramethylethylenediamine (TEMED), dopamine (DA), uric acid (UA), and ascorbic acid (AA) were purchased from J&K Scientific Ltd. (Beijing, China). Carboxymethyl chitosan (CMC, degree of substitution greater than 80%), hydrogen peroxide (H_2_O_2_, 30% aqueous solution), disodium hydrogen phosphate (Na_2_HPO_4_), and sodium dihydrogen phosphate (NaH_2_PO_4_) were obtained from Beijing Chemicals Co., Ltd. (Beijing, China). Natural graphite flakes (99.8% purity) were purchased from Sigma-Aldrich (Milwaukee, WI, USA).

### 2.2. Synthesis of GO, CMC/rGO, and DN Hydrogels

GO was synthesized according to a reported procedure with the Hummers’ method [20,21,22]. In brief, 3 g of graphite flakes and 18 g of KMnO_4_ were added to a H_2_SO_4_/H_3_PO_4_ (*v*/*v*, 9:1) solution in sequence. Then, 10 mL 10% H_2_O_2_ was slowly added to the mixed solution. Afterwards, the mixture was filtered, and the obtained yellow solid was washed with deionized water, ethanol, and HCl (30%), and precipitated with diethyl ether. Upon freeze-drying, 6.00 g GO was obtained.

CMC/rGO was synthesized by the chemical reduction of GO with CMC. In brief, GO was dissolved with deionized water at a concentration of 1 mg/mL. Then 0.03 g CMC was added into 2 mL GO (1 mg/mL) solution, and this reaction proceeded at 50 °C for 12 h with continuous stirring. 

To obtain DN hydrogels, 0.226 g NIPAm was added into the obtained CMC/rGO solution, along with 0.002 g BIS as cross-linking agent. After that, 1.1 mg APS as the initiator and 1 μL TEMED as the accelerator were added into the mixed solution. Finally, the mixture was reacted at 25 °C for 6 h in a nitrogen environment, and the PNIPAm/CMC/rGO DN hydrogels were prepared.

### 2.3. Compressibility of Hydrogels

Cylinder-shaped hydrogels (φ10 × 15 mm) were used in the compression tests. The mechanical analysis was carried out on a Lloyd LR30K tensile testing machine (Lloyd Instrument, Fareham, UK). A cross-head speed of 0.4 mm/min and a maximum strain of 60% were applied for the compression tests. At least three samples were evaluated in these compression tests in order to obtain reliable data. 

### 2.4. Pressure and Temperature Sensing

Cylinder-shaped hydrogels (φ10 × 20 mm) were chosen for these pressure sensing tests. The mechanical analyses were carried out on a Lloyd LR30K tensile testing machine, and the resistances were detected by a modified multimeter. The resistance changes of the hydrogels were monitored. In order to improve the contact surface and measurement stability during the pressure sensing tests, the probe head of the multimeter was shaped into a pointed needle with a flat elliptical surface. 

The formed hydrogel samples were placed on a heating stage and the temperature was continuously adjusted. The resistance changes were detected by the modified multimeter. It was important to make sure that the probe did not touch the heating table. Then, the sample was covered with a transparent cover. The changes of resistance of the formed hydrogels when exposed to different temperatures were recorded. The experiments were performed at least three times for statistical analysis.

### 2.5. Characterization Techniques

Ultraviolet-visible (UV–Vis) analyses were performed by using a Lambda 365 spectrometer (scanning rate 240 nm/min, Perkin-Elmer, Waltham, MA, USA). The Fourier transform infrared (FTIR) spectra of the samples were recorded by a Nicolet 6700 spectrometer (Thermo-Fisher, Waltham, MA, USA). Transmission electron microscopy (TEM) images were obtained with a TH7700 microscope (JEOL, Tokyo, Japan) with an accelerating voltage of 200 kV. Atomic force microscopy (AFM) images were acquired with a NanoWizard 3 atomic force microscope (JPK Instruments, Berlin, Germany). Scanning electron microscopy (SEM) images were obtained with a JSM-6700F microscope (JEOL, Tokyo, Japan). The resistances were detected by a modified multimeter whose probe head was shaped into a pointed needle with a flat elliptical surface.

## 3. Results and Discussion

### 3.1. Characterizations of GO and CMC/rGO

**Figure 1:**
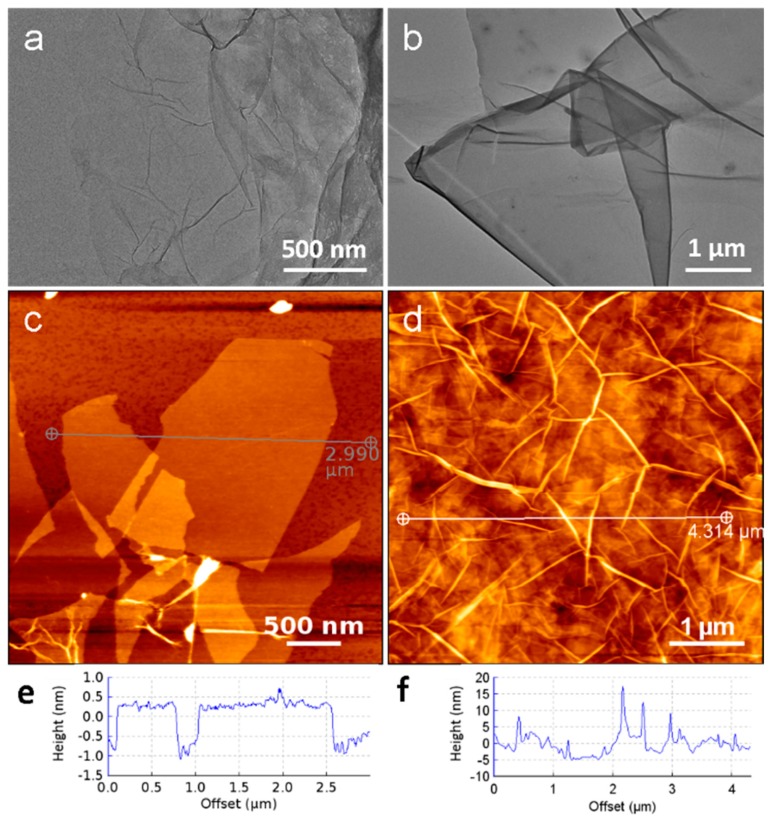
Transmission electronic microscopy (TEM) image of (**a**) graphene oxide (GO) and (**b**) carboxymethyl chitosan (CMC)/reduced GO (rGO). Atomic force microscopy (AFM) images and corresponding section analyses of (**c**,**e**) GO and (**d**,**f**) CMC/rGO.

The morphologies of GO and CMC/rGO were first characterized by TEM and AFM, and the results are shown in Figure 1. A TEM micrograph of GO nanosheets is shown in Figure 1a. It is clear that the GO nanosheets exhibited an extended thin film with a wrinkled surface, which prevented the collapse of GO back into a graphite structure [23]. The cross-sectional view towards the AFM image of the exfoliated GO (Figure 1c,e) shows that GO presented an ultrathin lamellar structure with a height of 0.5–1 nm, which is consistent with the reported thickness of GO in previous AFM studies [24]. 

CMC with amino groups was introduced to reduce hydrophilic GO, and thus improve the conductivity of the hydrogels [25]. TEM images showed the presence of dark patches after the combination of GO with CMC (Figure 1b). We suggest that the increased thickness of the graphene sheets indicates that GO nanosheets were covered by CMC. In addition, both wrinkles and a closed layer structure can be seen in the obtained AFM image (Figure 1d). The observed morphology of CMC/rGO can be ascribed to the formation of amide bonds between the amino groups of CMC and the oxygen groups of GO [26,27]. 

**Figure 2:**
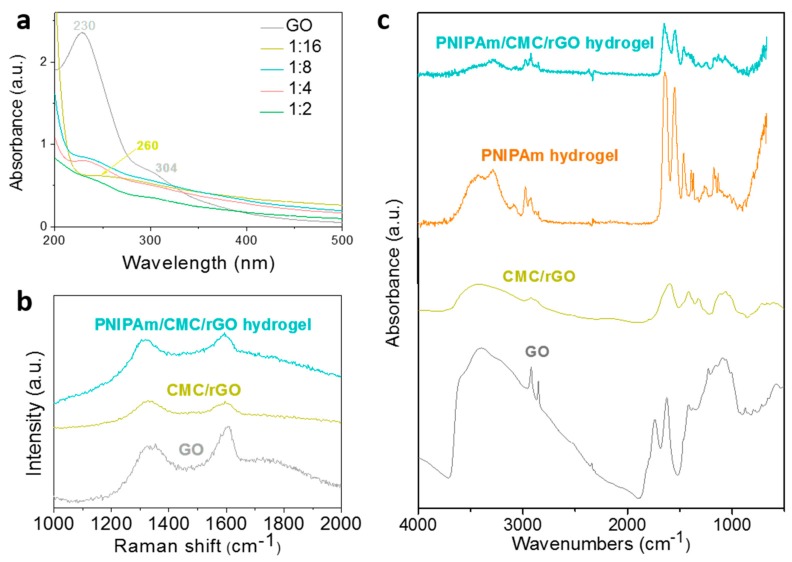
Structure characterizations of GO, CMC/rGO, and poly(*N*-isopropylacrylamide) (PNIPAm)/CMC/rGO hydrogel: (**a**) UV–Vis absorption spectra of GO and CMC/rGO with different CMC additions by adjusting GO/CMC to 1/16, 1/8, 1/4, and 1/2; (**b**) Raman spectra; and (**c**) Fourier transform infrared (FTIR) spectra.

The effect of CMC concentration on the reduction efficiency of CMC/rGO was investigated. Various ratios GO to CMC (GO/CMC; e.g., 1/0.5, 1/1, 1/2, 1/4, 1/8, 1/16, and 1/32) were designed and studied. As the ratio decreased, the color of the CMC/rGO solution gradually changed from light yellow to dark black, indicating that GO was reduced effectively. However, when the ratio of GO/CMC was 1/32, the solution exhibited poor solubility. UV–Vis absorption spectra showed that the absorbance strength of CMC/rGO increased as the CMC content increased (Figure 2a). Considering the reducibility and solubility of CMC/rGO, the ratio GO/CMC 1/16 was selected for follow-up experiments. The intense peak at a 230 nm wavelength is the characteristic peak of GO, which is related to the π–π* transition of C=C [28]. The small shoulder at a 304 nm wavelength relates to the n–π* transition of C=O [29]. After the addition of CMC, the peak at 230 nm shifted to 260 nm, suggesting the successful reduction of GO. The shift of the UV–Vis absorption peak is ascribed to the restoration of both electronic conjugation and sp^2^ graphitic carbons within graphene sheets after a chemical reaction [30]. 

Raman analysis was applied to investigate the GO layers and the CMC attachment on GO (Figure 2b). The characteristic peaks of GO in the Raman spectrum from 1000 to 2000 cm^−1^ are related to D and G bands located at 1358 and 1595 cm^−1^, respectively [31,32]. The G band of single-layered GO and multi-layered GO (2–6 layers) were at 1585 and 1594 cm^−1^, respectively. Here, the D- and G-band peaks of GO were located at 1326 and 1597 cm^−1^, respectively. After reduction by CMC was performed, the peaks of GO remained at the same position, which indicates that multilayered GO sheets were synthesized and retained the same structure. In addition, the intensity ratio of D to G band (I_D_/I_G_) can be used to assess the reduction degree of rGO. In this work, the value of I_D_/I_G_ changed from 0.7 to 1.0, indicating the attachment of CMC onto GO, and the successful reduction of GO to rGO. The size of D- and G-band shifts and the change of the I_D_/I_G_ ratio revealed that GO was successfully functionalized with CMC.

The FTIR spectra in Figure 2c show the functional groups of GO, which exhibit rich oxygen-functional groups. The existences of O–H, C=O, and C–O groups of GO were confirmed by the absorption peaks at 3402, 1740, and 1090 cm^−1^, respectively. As for CMC/rGO, most of the vibration peaks of GO and CMC/rGO were overlapped. The peak of C=O groups disappeared, and the peak intensities of other oxygen-functional groups noticeably decreased. The obtained results proved that the carboxylic groups of GO reacted with the NH_2_ groups in CMC. Based on the above FTIR results, we suggest that CMC was successfully conjugated onto rGO.

### 3.2. Morphological Characterizations of PNIPAm/CMC/rGO DN Hydrogels

After the integration of CMC/rGO hybrids into PNIPAm hydrogels, the structure properties of the fabricated DN hydrogels were further characterized by Raman and FTIR (Figure 2b,c). The typical peaks of GO in Raman spectra remained at the same position, and the I_D_/I_G_ ratio was at 1.0. In the FTIR spectra, the characteristic peaks of PNIPAm could be observed, even though some of them were overlapped by the spectrum of CMC/rGO nanohybrids. It is suggested that the PNIPAm/CMC/rGO DN hydrogels were successfully prepared.

**Figure 3:**
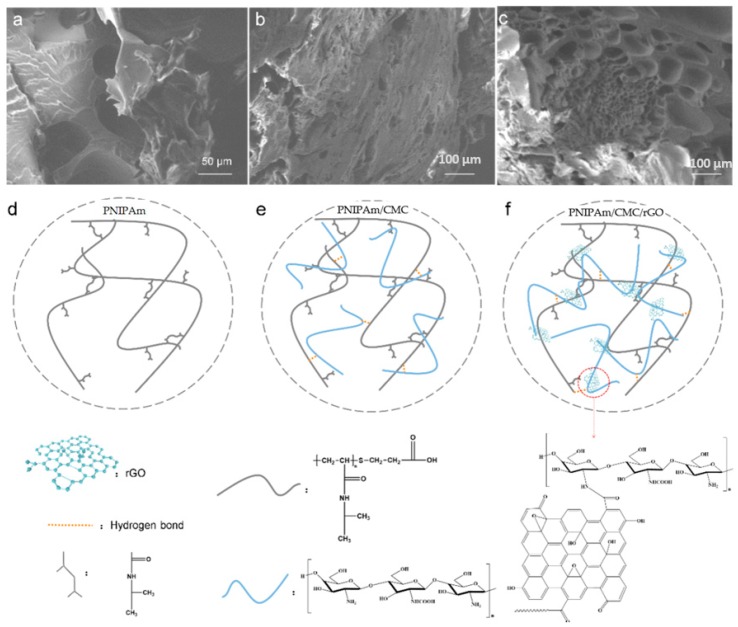
SEM images of (**a**) PNIPAm; (**b**) PNIPAm/CMC; and (**c**) PNIPAm/CMC/rGO hydrogels. (**d**–**f**) Schematic representation of the (**d**) PNIPAm; (**e**) PNIPAm/CMC; and (**f**) PNIPAm/CMC/rGO hydrogels.

The SEM image of the PNIPAm hydrogels displays a homogeneous polymer surface without micropores (Figure 3a). The PNIPAm hydrogel had high cross-linking density. While for the PNIPAm/CMC hydrogel, a porous structure appeared on the surface due to the addition of CMC molecules (Figure 3b). With the addition of CMC molecules, the cross-linking sites in the PNIPAm/CMC hydrogels decreased, and the cross-linking density of the hydrogels appeared to be lowered. Furthermore, when the DN was formed in the PNIPAm/CMC/rGO hydrogels, porous structures with large pore size were created (Figure 3c). The micropores in the DN hydrogels exhibited a more uniform shape and were more obvious than those of PNIPAm/CMC hydrogels. This phenomenon can be attributed to the integration of GO and DN in the PNIPAm/CMC/rGO hydrogels. 

Figure 3d–f represent the possible network structures of the three types of hydrogels. In the PNIPAm hydrogel, NIPAm molecules lead to densely packed cross-linking networks [33]. In the PNIPAm/CMC hydrogel, the CMC molecules hindered the formation of hydrogel and led to a porous network structure. With the addition of CMC/rGO into the PNIPAm hydrogel, a DN hydrogel was fabricated. One network comes from the cross-linked NIPAm molecules, and the other originates from the connection of CMC molecules to GO. The CMC molecules are connected via an aminocarbonyl reaction onto GO nanosheets. Additionally, the hydrogen bonds between PNIPAm and CMC molecules promote the formation of DN structure. 

### 3.3. Compressibility and Pressure Sensing

**Figure 4:**
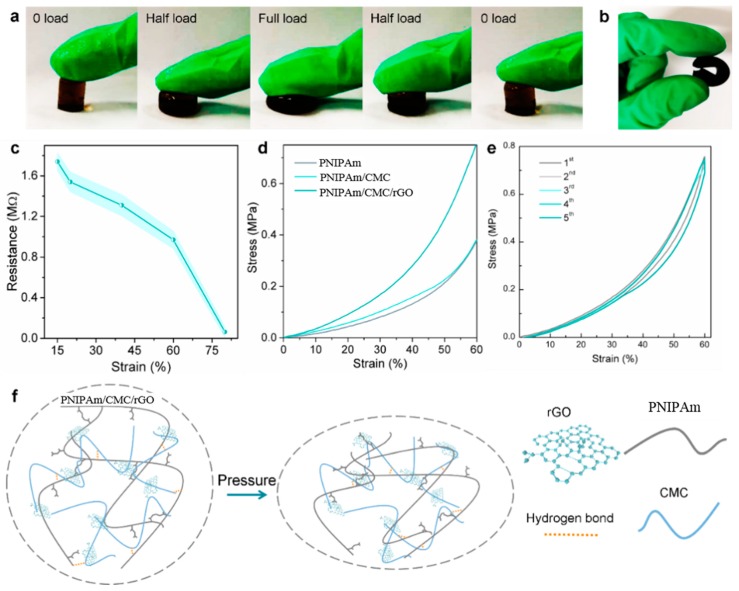
(**a**) Compressibility and (**b**) bendability of PNIPAm/CMC/rGO hydrogels. Pressure sensing of PNIPAm/CMC/rGO hydrogel: (**c**) Resistance-strain curves (the light blue bands are the error bars for the values); (**d**) Compression stress–strain curves; and (**e**) cyclic compression stress–strain curves of PNIPAm/CMC/rGO hydrogels. (**f**) Schematic drawing of the compression process.

Unlike the traditional PNIPAm hydrogels which are brittle and rigid [34], the PNIPAm/CMC/rGO DN hydrogels showed good compressibility and flexibility (Figure 4a,b). The as-prepared hydrogels could be compressed to a great extent, and the original shape returned after the pressure was released. When the hydrogels present a sheet structure, they could also be bent as easily as rubber, indicating that the PNIPAm/CMC/rGO hydrogels were flexible as elastic materials. 

Moreover, the stress–strain curves of hydrogels were analyzed, as shown in Figure 4d. The PNIPAm hydrogel and the PNIPAM/CMC hydrogel exhibited the compression strengths 0.376 MPa and 0.378 MPa, respectively. A higher compression strength (0.754 MPa) was obtained by the PNIPAm/CMC/rGO hydrogels without damage, which indicates that the compression property of the PNIPAm/CMC/rGO hydrogels was approximately double that of PNIPAm and PNIPAm/CMC hydrogels. 

In addition, the cyclic compression of the PNIPAm/CMC/rGO hydrogels was studied, and the results are shown in Figure 4e. It was found that the formed PNIPAm/CMC/rGO hydrogels had good compressibility and recyclability, and could endure an infinite number of stress cycles. As for the PNIPAm hydrogel and PNIPAm/CMC hydrogels, cracks occurred within 5 cycles. The above data confirms that the PNIPAm/CMC/rGO hydrogels displayed better cyclic compressibility. 

The pressure sensing characteristic of the PNIPAm/CMC/rGO hydrogels was investigated. Figure 4c shows a typical plot of relative variation in resistance as a function of applied strains. The hydrogels exhibited a continued decrease in resistance when the maximal strain reached 80%, which may indicate that the continuous layer structure of GO and the tight DN structure of hydrogels improved the continuous electronic transmission, and thus resulted in pressure sensing.

We suggest that the deformation process of the hydrogel consists of two steps. In the first step (lower than 40% strain), the modulus increases steadily with the stress. Due to the stretch of the PNIPAm network in the vertical direction, the deformation is perpendicular to the compression direction. In this way, the energy absorption can enhance the mechanical properties of PNIPAm/CMC/rGO hydrogels. In the second step (higher than 40% strain), the modulus increases drastically with the stress. This is likely attributable to the stretch of the second network of CMC/rGO in the vertical direction and the breaking of the hydrogen bonds between the two networks. This unique network structure of the composite hydrogels showed a high pressure response, and exhibited sensitive and stable resistance–compression sensitivity.

### 3.4. Temperature Sensing

**Figure 5:**
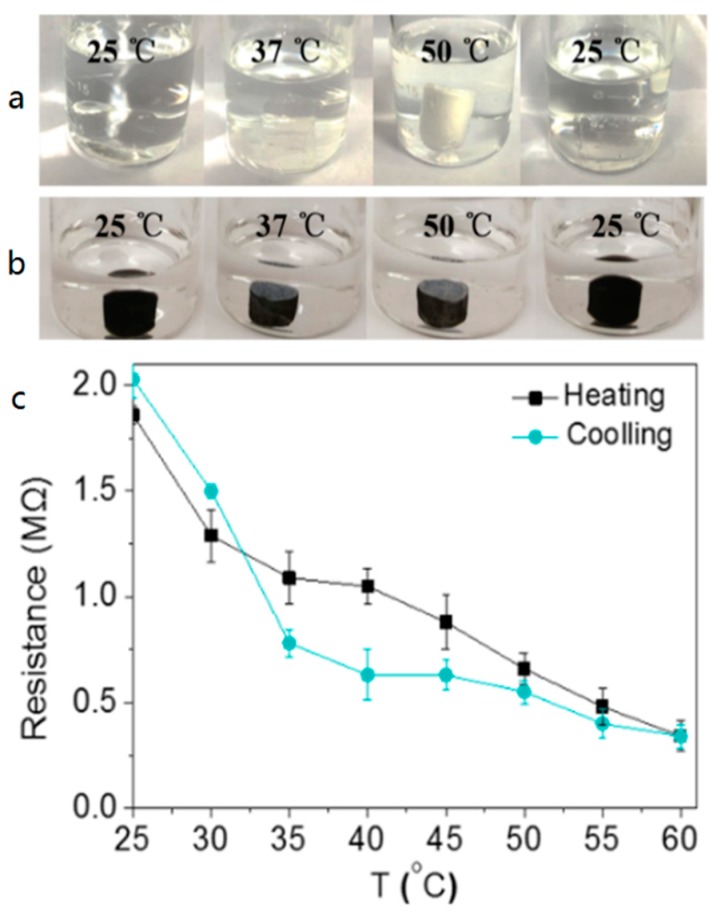
The thermosensitivity of hydrogels: (**a**) PNIPAm; (**b**) PNIPAm/CMC/rGO; (**c**) Resistance-temperature cycling curves of PNIPAm/CMC/rGO hydrogels.

PNIPAm endowed the PNIPAm/CMC/rGO hydrogels with excellent thermosensitivity. GO was reduced by the amino groups of CMC, which thus improved the conductivity of the fabricated hydrogels. All these properties are helpful to develop skin-like devices for temperature sensing. It was found that both PNIPAm and PNIPAm/CMC/rGO hydrogels exhibited excellent thermosensitivity, as shown in Figure 5a,b. The PNIPAm hydrogels were transparent, with swelling below the critical temperature (32 °C) and diffusing above this temperature, while the colour of the PNIPAm/CMC/rGO hydrogels did not noticeably change with the temperature due to the additional colour effect of graphene nanosheets.

Previously, some of the reported materials showed a basic temperature sensitivity, but did not allow for temperature sensing [35]. It should be noted that the created PNIPAm/CMC/rGO DN hydrogels displayed cyclic resistive changes (1.86–0.34–2.03 MΩ) during repeated heating and cooling (Figure 5c), due to their thermosensitivity and conductivity. During the cooling process, the resistance of hydrogels basically recovered its original value, indicating that the temperature sensing of the hydrogels had good stability and repeatability. Moreover, the temperature sensing could be realized without any external stimulus (e.g., UV light) [36,37]. Therefore, the created DN hydrogels could be used to produce nano/micro-devices for detecting the temperature of the human body.

## 4. Conclusions

In summary, novel DN hydrogels, PNIPAm/CMC/rGO, were designed and developed using a facile procedure. The rGO integrated in the hydrogels, as well as the combination of dense PNIPAm networks and ductile CMC/rGO networks, improved the compressibility and flexibility of the PNIPAm/CMC/rGO DN hydrogels to a great extent. The first network provided sacrificial bonds and dissipated energy, whereas the second network endowed the hydrogels with elasticity and conductivity. The fabricated DN hydrogels exhibited good conductivity, temperature-sensitivity, and compressibility, which allowed both pressure and temperature sensing with a wide resistance change. These findings proved the potential applications of rGO-based DN hydrogels in multiple sensing fields.
